# Neural Correlates of Public Apology Effectiveness

**DOI:** 10.3389/fnhum.2019.00229

**Published:** 2019-07-25

**Authors:** Hoh Kim, Jerald D. Kralik, Kyongsik Yun, Yong-an Chung, Jaeseung Jeong

**Affiliations:** ^1^Graduate School of Culture Technology, Seoul, South Korea; ^2^Department of Bio and Brain Engineering, Korea Advanced Institute of Science and Technology (KAIST), Daejeon, South Korea; ^3^Department of Radiology, College of Medicine, The Catholic University of Korea, Seoul, South Korea

**Keywords:** conflict resolution, crisis management, fMRI, causal attribution theory, social neuroscience

## Abstract

Apologizing is an effective interpersonal conflict resolution strategy, but whether, and if so how, organizations should issue public apologies after crises remains less clear. To assuage the fear of possible crisis reoccurrence, public apologies may be effective when they provide a comprehensive account of what happened and clarify actions taken by the company to address the problems. If this is so, public apologies may be most effective when the crisis source resides within the organization itself, suggesting that the company has control over it. In the current study, we first tested this hypothesis by presenting participants with multiple crisis scenarios (e.g., ignition failures in a new car model) followed by one of two written apologies: one stating that the crisis source was internal to and controllable by the organization, and the other external and uncontrollable. The internal-controllable (IC) public apology proved most effective. We then examined the neural basis of this public apology assessment and found that the frontal polar cortex appears to mediate the assessment of organizational control, and the angular gyrus uses the information for the apology assessment. Examination of complex social interactions, such as the public’s reaction to corporate crises, helps to elucidate high-level brain function.

## Introduction

With repeated social interaction, conflict is inevitable, and one of the oldest known forms of conflict resolution is for the harmdoer to apologize (Goffman, 1971; Benoit, 1995; Lazare, 2004). A great deal of evidence has shown that interpersonal apologies can be effective (Tomlinson et al., 2004; Zechmeister et al., 2004; Frantz and Bennigson, 2005; Anderson et al., 2006; Tucker et al., 2006; Boothman et al., 2009), however, with increasingly more complex social interactions the results are less clear. Thus, when larger organizations such as corporations are involved in crisis events, they must determine whether to issue a public apology, and if so, what to say. Yet with the dearth of evidence for public apology effectiveness, as well as some evidence for actual ineffectiveness (Shaw et al., 2003; Kim et al., 2004, 2015; Philpot and Hornsey, 2008; Page, 2014), many corporations and their advisors in fact believe that too much information may be more harmful than not. Complicating matters, of course, is the potential legal and economic ramifications of declaring responsibility or otherwise upsetting the public even further. This belief has led companies into issuing vague and sometimes misleading public statements, such as in the recent case of United Airlines forcefully removing a customer from the plane, which was recorded by passengers and posted online. The company was forced to issue a series of progressively more forthright responses, but the damage had been done (Creswell and Maheshwari, 2017; Grynbaum, 2017; McCann, 2017).

In fact, a prominent public concern is the potential threat of reoccurrence (Shaw et al., 2003; Philpot and Hornsey, 2008; Page, 2014; Kim et al., 2015). And given this, one might expect that a public statement that clearly described the reasons for the crisis and how the company can prevent the reoccurrence would assuage public fears of a repeated offense, and thus be particularly effective. From the public’s perspective, they must assess the likelihood the company will be able to eliminate or control the crisis source to prevent future problems. To assess this likelihood of control and prevention, which we call *control assessment*, two factors are particularly relevant: (1) whether the company actually has the ability to control the source (i.e., one can see a direct causal link between them), i.e., *controllability*; and (2) the relative balance of the organization vs. other possible factors that can influence the crisis source, i.e., the degree of the organization’s *exclusivity* of control. Factor (2), exclusivity, takes into account the relative prevalence of other factors that also have potential controllability over the crisis source, which could possibly counteract or somehow negate the company’s potential influence. Thus, for example, if the crisis source is found to be *internal* to the organization, the organization should have relatively exclusive control over the source; whereas, if the crisis source is *external* to the organization, other factors may possibly influence it, which may be outside the organization’s purview, planning, and control. Thus, having the source of the crisis as both internal to the organization (i.e., relatively exclusive influence), and controllable would together provide the strongest case for organizational control over the causal factors.

Note, however, that this line of reasoning leads to a perhaps counterintuitive prediction: the public may prefer that the company actually caused the crisis. Some evidence for this counterintuitive prediction was obtained by Lee et al. (2004) who examined shareholder reactions to corporate annual reports. Companies that explained poor performance as being due to internal, controllable causes (e.g., some internal malfunction) as opposed to causes outside of their control (e.g., unpredictable downturn in economy) were found to have higher stock prices the following year, presumably reflecting not only future company performance, but stockholders’ assessments of it as well. Thus, company statements that suggest organizational control over crisis causes and thus effective control over future events appear to produce a more favorable impression. More broadly, the study’s findings may suggest that the potential threat of crisis reoccurrence is particularly important to people, with the public attempting to assess future events *via* their evaluation of the actual causal factors of the current crisis and the likelihood that they will be controlled by the organization. The findings might further imply that effective public statements require significant disclosure of crisis details, enabling proper assessment of the crisis and the organization by the public. To be sure, following the United Airlines crisis involving the forceful removal of the passenger, public furor could not be stemmed until such disclosure was finally made by the company (Creswell and Maheshwari, 2017; Grynbaum, 2017; McCann, 2017).

Given the importance of future threat assessment, as well as supporting evidence that suggests people need to assess the causal factors underlying the crisis, in the current study we attempted to test this hypothesis broadly across a range of corporate crises. We then examined the neural correlates of the public apology assessments, particularly focusing on identifying the brain regions underlying crisis control assessment and the use of this information to assess the public apology.

Although reconciliation and the offering of apologies to aggrieved parties is an ancient form of conflict resolution (with the former found in chimpanzees and thus other hominids as well; de Waal, 1990), to date, very little is known about the neural correlates. With respect to interpersonal apologies, one study obtained some initial results of the neural mechanisms underlying the receiving of an apology (Strang et al., 2014). Potential transgressors were given a multiple-choice test (e.g., “What is the capital of Norway?”) and their answers led to payoffs for both themselves and someone else (the receiver). When answers were incorrect, the payoff was *increased* for the transgressor and *reduced* for the receiver, providing incentive to answer incorrectly. However, because questions were moderately difficult (itself leading to both correct and incorrect answers), potential wrongdoing was ambiguous. Nonetheless, those answering were allowed to make an apology when they were incorrect (which if accepted increased the answerer’s payoff). In this interpersonal apology situation, receiving an apology (vs. not receiving one) produced increased activity in the left inferior frontal gyrus (and more specifically, orbitofrontal cortex), the left middle temporal gyrus, and the left angular gyrus of the receiver. When forgiving (vs. not forgiving), increased activity was found in the right angular gyrus of the receiver. With respect to these activated regions, given that they have also been implicated in tests of empathy (i.e., sharing another individual’s emotional states), and given that other behavioral studies suggest a possible causal chain from apology to empathy to forgiveness (McCullough et al., 1998), the authors concluded that the neural evidence suggests that the apologies evoked empathy, i.e., an appreciation of the transgressor’s point-of-view.

Another functional imaging study compared costly apologies (e.g., treating someone to lunch after missing previous appointment) vs. non-costly ones (e.g., simply saying sorry) (Ohtsubo et al., 2018). They found activation in brain regions normally associated with mentalizing (i.e., consideration of the mental states of others, also called theory of mind, and typically distinct from empathy, with the former focused more on cognitive versus emotional aspects of the other’s mind): i.e., medial prefrontal cortex (MPFC), bilateral temporoparietal junction (TPJ), and precuneus.

Nonetheless, multiple factors remain unknown, such as how specific content elements are processed (e.g., crisis event factors or other components of the apology), how they lead to apology effectiveness, and the extent to which these findings generalize to other cases, such as intergroup crises and public apologies. Indeed, to our knowledge, no study to date has examined the neural mechanisms underlying effective *public* apologies. Finally, because our study highlights the concerns people have about crisis reoccurrence, and the importance of assessing the causal attributions (i.e., the specific crisis cause and the organization’s potential control over it), we examined the underlying neural mechanisms of this type of causal analysis.

## Materials and Methods

To measure apology effectiveness, we asked participants to rate *account acceptance* (“The people affected by the incident would consider the response by the organization to be appropriate.”; Coombs and Holladay, 2008). We selected 11 short news articles that provided a range of corporate crises (on average, 60.27 words, 245.09 characters in Korean language including spaces; see Tables s 1 and 2). For each article, we provided two possible public apology statements: one using the *internal-controllable* (IC), and the other the *external-uncontrollable* (EU) attribution. With respect to the crisis cause being internal vs. external to the organization, i.e., *exclusivity*, we focused on whether the causal actions leading to the wrongdoing were part of the normal operating procedures of the company (versus, for example, being due to personal self-interested motives). Thus, the wrongdoer could be an employee of the organization, but whether the wrongdoing was considered internal or external depended on the actions taken. In addition, there are two types of causal actions that should both be considered internal to, and thus the direct result of, the organization: (1) actions taken as part of the standard operating practices of the organization that directly produced the harmful event; or (2) actions *not taken* that should have been performed as part of standard operating procedures that if performed would have prevented the harmful event. Cases (1) or (2) were considered *internal*, otherwise, they were considered *external*. 1 provides an example of Case (1), and Table 2 provides an example of Case (2).

**Table 1:** Example scenario and the public apologies used for the internal-controllable and external-uncontrollable attribution conditions.

	Regret	External/Internal	Uncontrollable/Controllable	Repetition and Closing
External/Uncontrollable	Apology from OO Motors: we are truly sorry for the recent controversy spread across the web and in the media regarding our latest model OO.	An internal investigation by OO Motors has confirmed that the problem was caused by quality issues at our outsource partner that has been supplying parts for several of the 2010 models. EXTERNAL: blaming “outsource partner”	The failure was a result of a part manufactured by an outsource partner, therefore, it was hard for us to take preventive measures.	OO Motors is committed to always strive for customer safety.
Internal/Controllable		An internal investigation by OO Motors has proven that there were problems with some of the parts used in the 2010 models. INTERNAL: no suggestion that the cause was external	As a company that should have been responsible for final quality assurance, we have failed to thoroughly control the process.	OOO CEO of OO Motors

*Faulty ignition leads to a flood of complaints against OO Motors. OO Motor’s latest model launched earlier this year was found to have problems with ignition caused by defects in its electric cabling. However, OO Motors has been keeping quiet about the ignition failures while only fixing it for customers who had complained leading to a potential class action started online. Meanwhile, OO Motors rebutted the fact claiming it was just a common problem. Consumer complaints are unlikely to dissipate for a while (Industrial News, Kim Hyung-Kyun)*.

**Table 2:** A second sample crisis event and the public apologies used for the internal-controllable and external-uncontrollable attribution conditions.

	Regret	External/Internal	Uncontrollable/Controllable	Repetition and Closing
External/Uncontrollable	OO Bank’s Apology: we deeply apologize for disappointing our customers with the recent embezzlement case.	It has been confirmed that the employee accused of the recent embezzlement case acted entirely on his own. EXTERNAL: “acted entirely on his own”	OO Bank had no way of knowing about this case. Hence, OO Bank was limited in what it could have done to prevent this from happening.	We are committed to implementing more thorough ethical training and rigorous supervision to prevent such a case from reoccurring. OOO
Internal/Controllable		The recent embezzlement case was a result of a carefully planned crime by an employee of OO Bank caused by poor employee management. INTERNAL: blaming organization by saying “caused by poor employee management”	Therefore, OO Bank should have prepared stricter measures to supervise its employees and ensure this kind of case never occurs.	CEO of OO Bank

*00 Bank director flees after embezzling over KRW 12 billion from customers. According to police investigations, a director in the private banking division of OO bank identified by the last name Kim (51 years old) was found guilty of fleeing after misappropriating roughly KRW 12 billion from customers’ savings accounts. Seoul Nambu Police Agency has issued a search warrant for the suspect and launched a probe against all employees for possible accomplices in the bank. Customers of Kim have been making complaints to the bank in the wake of the news (Seoul, Yoon Kyung-Shik)*.

Thus, while in the functional magnetic resonance imaging (fMRI) scanner, each participant was exposed to 11 news articles randomly presented, and after each news article, one of the two public apology statements, also randomly presented, and they rated the appropriateness of the apology using a Likert-type five-point scale. Methods details are as follows.

### Participants

We recruited 42 adults (22 females), and the data from two female participants were discarded for technical problems. Screening procedures were used (i.e., lived nearby, Korean and could read Korean well, ages 20 s–50 s, high school graduate or above, right handed, not claustrophobic or pregnant or in the process of breast-feeding, no metal in their body, not currently taking a psychoactive drug), and we received written informed consent from each participant. The Catholic University School of Medicine Review Board and the Korea Advanced Institute of Science and Technology (KAIST) Review Board approved the study.

### Materials

We used 11 mock news reports based on real crisis events (see Tables s 1 and 2), along with the two apologies created for each based on the external/uncontrollable and internal/controllable attributions. We then asked the participants to rate one statement regarding the appropriateness of the response using a five-point Likert scale: “The people affected by the incident would consider the response by the organization to be appropriate.” Responses were obtained using a four-button response panel; if participants chose a five rating, they pushed the fourth button twice.

### Experimental Protocol

The crisis event news reports were presented randomly, followed by one of the two public apologies randomly selected, *via* a visual display projected into the scanner. Each trial consisted of six screens: a news report as text on the first screen (duration: 20 s), then a public apology statement with either an internal/controllable or external/uncontrollable attribution as text through a series of four screens [for each component of the apology as seen in Tables s 1 and 2: regret (duration: 6 s), internal or external crisis source (6 s), controllable or uncontrollable (6 s), then statement to prevent reoccurrence (6 s)], and the last screen asking the appropriateness of the response using a five-point Likert scale (6 s). During the intertrial interval, a fixation cross was viewed for 6 s, followed immediately by the subsequent trial.

### fMRI Data Acquisition

Functional imaging was conducted using a 3.0 Tesla Trio magnetic resonance imaging (MRI) scanner to acquire gradient echo T2*-weighted echoplanar (EPI) images with blood oxygenation-level-dependent (BOLD) contrast. Each volume of images had 32 axial slices. The imaging parameters were as follows: echo time, 27 ms; field of view, 192 mm^2^; in-plane resolution and slice thickness, 4 mm (no gap); repetition time, 2 s. Whole-brain high-resolution T1-weighted structural scans (0.9 × 0.9 × 0.9 mm^3^) were acquired for each participant, co-registered with their mean EPI images and averaged across participants to permit anatomical localization of the functional activations at the group level.

### fMRI Data Analysis

Image analysis was performed using SPM8 (Wellcome Department of Imaging Neuroscience, Institute of Neurology, London, UK). We corrected the images for slice acquisition time within each volume and for motion artifact with realignment to the first volume. We also spatially normalized images to the standard Montreal Neurological Institute EPI template and spatially smoothed the images using a Gaussian kernel with a full width at half maximum of 8 mm. Intensity normalization and high-pass temporal filtering (using a filter width of 128 s) were also applied to the data. We estimated each participant-level general linear model (GLM) using a first-order autoregressive model. The purpose of this model was to identify the regions related to the EU and IC conditions, as well as those correlated with the behavioral ratings of apology appropriateness. Analysis was conducted during the 6-s “controllable or uncontrollable” display (which always followed the external/internal condition). The regressors included the two trial types (EU and IC), and the participants’ rating value in the trial. Motion parameters and session constants were included as regressors of no interest. We calculated contrasts for the difference between the parametric regressor for the internal/controllable condition and that for the external/uncontrollable condition. In addition, we examined the correlation between brain activity and rating scores in both the IC and EU conditions separately.

The contrasting images computed for each participant were taken to the group random effects level. We computed the contrasts between the IC/EU conditions, as well as examined the correlation between brain activity and rating scores in both the IC and EU conditions separately. We used the false discovery rate (FDR) correction for multiple comparisons with a threshold of *p* < 0.05.

## Results

### Behavioral Findings

**Figure 1:**
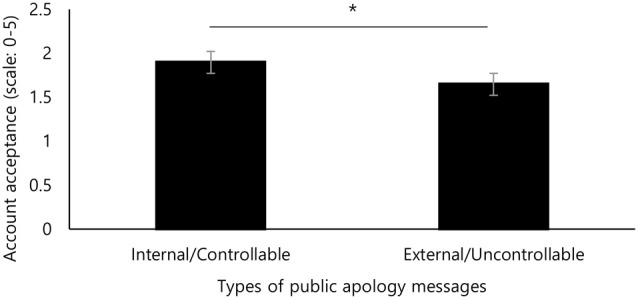
Public apologies with an internal/controllable attribution were more effective than those with an external/uncontrollable attribution in terms of account acceptance. Error bars are standard error of the mean (SEM). *p < 0.05.

As seen in Figure 1, account acceptance was significantly higher for the internal/controllable (IC) condition than for the external/uncontrollable (EU) one (*t*_(39)_ = 2.15, *p* = 0.038). Thus, we found that even without necessarily admitting wrongdoing, the public apologies proved most effective when the source of the crisis was both internal to and controllable by the organization. The results support the hypothesis that apologies would be more persuasive when they clearly demonstrate having the power to rectify the problem and limit possible reoccurrence. Moreover, although the internal and controllable attribution may not mean the acceptance of full crisis responsibility, it is also likely that it is seen as a responsible gesture, which may also contribute to effectiveness.

### Imaging Findings

**Figure 2:**
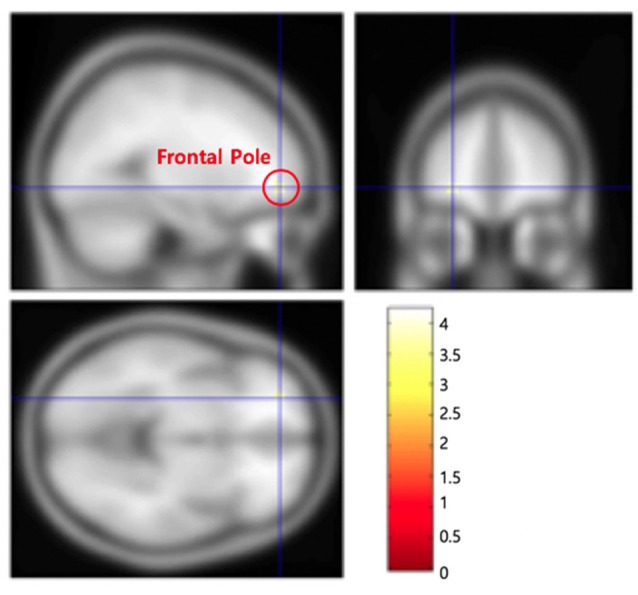
Frontal polar cortex (−26, 50, −6) in the left hemisphere was significantly activated in the internal/controllable condition as contrasted to the external/uncontrollable condition.

**Figure 3:**
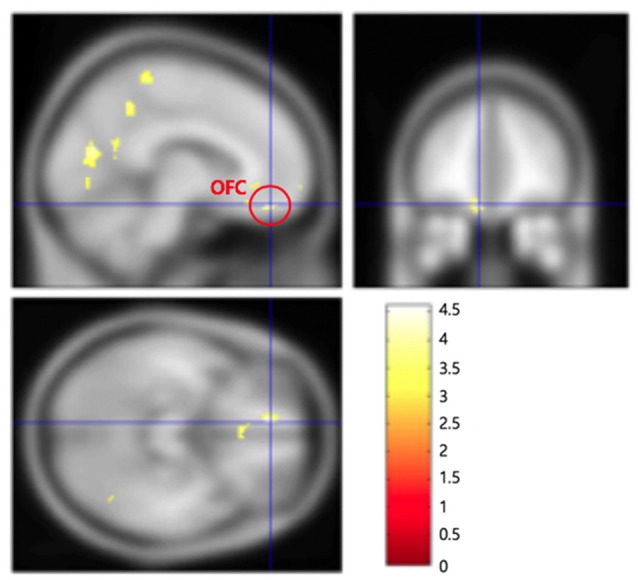
Orbitofrontal cortex (OFC; −8, 44, −18) in the left hemisphere was more activated in the external/uncontrollable condition than in the internal/controllable condition.

**Figure 4:**
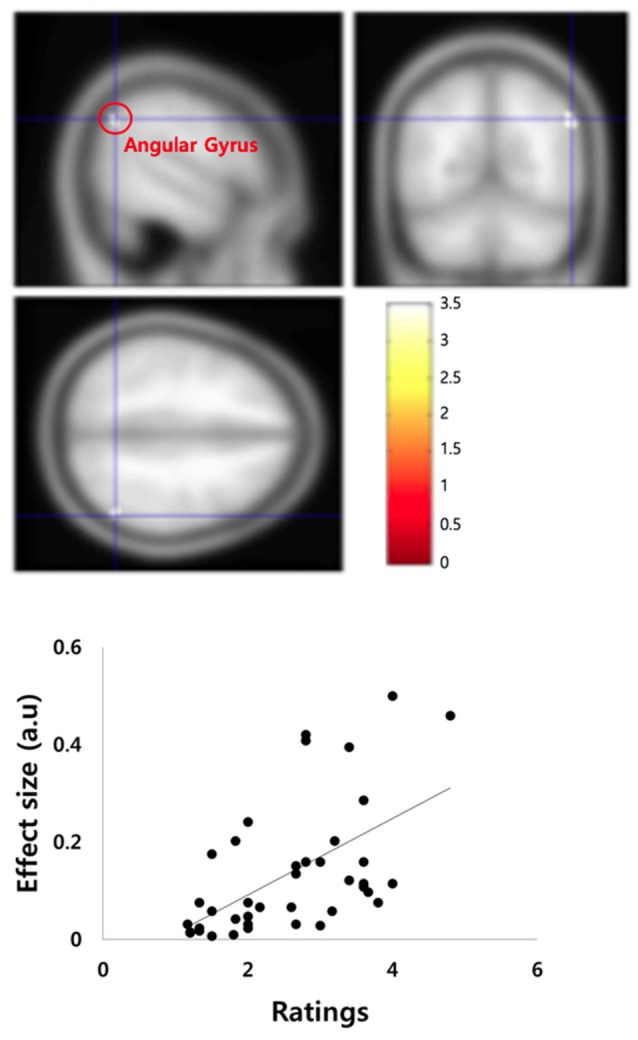
Parametric modulation found a significant correlation between angular gyrus activity (54, −60, 38) in the right hemisphere and the *account acceptance* ratings in the internal/controllable condition.

There were three main imaging results. First, frontal pole in the left hemisphere (left BA 10; −26, 50, −6, *z*-score: 3.33, *p* < 0.05, false discovery rate—FDR—corrected) was significantly activated in the IC condition as contrasted to the EU condition (Figure 2 and Supplementary Table S1). Thus, (left) frontal pole appeared to mediate the internal and thus organizational control assessment. Second, ventromedial prefrontal cortex (VMPFC), and more specifically, orbitofrontal cortex (OFC) in the left hemisphere (left BA 11; −8, 44, −18, *z*-score: 3.64, *p* < 0.05, FDR-corrected) was more activated in the EU condition than in the IC condition (Figure 3 and Supplementary Table S2). Thus, OFC appeared to mediate the external control assessment. Hence left frontal and ventral PFC appear to mediate causal attributions *via* a control assessment, with each subregion activated more for each end of the IC/EU spectrum. Third, we found a significant correlation between right angular gyrus activity (54, −60, 38, *z*-score: 3.31, *p* < 0.05, FDR-corrected) and the *account acceptance* ratings in the IC condition (Figure 4 and Supplementary Table S3). No brain regions showed significantly correlated activation with the *account acceptance* ratings in the EU condition. The relationship of the angular gyrus activity to the account acceptance ratings in the IC condition suggests that the angular gyrus mediated the effect of the organizational control assessment on the ultimate public apology assessment. Taken together, the imaging results suggest that the organizational control assessment in the left frontal PFC is transmitted to the angular gyrus, which in turn uses the information for the apology assessment.

## Discussion

### Behavioral Findings

The behavioral results support the hypothesis that public apologies are more effective when the public believes the company has identified the crisis cause and has the ability and purview to correct the problem and thus minimize possible reoccurrence. The results corroborate those found for corporate annual reports to shareholders, in which companies who explained poor performance as being due to internal, controllable causes (e.g., an internal error) as opposed to causes outside of their control (e.g., unpredictable suppliers) had higher stock prices the following year, reflecting more favorable stockholder assessments (Lee et al., 2004). Here, we have extended the findings to corporate crises more generally.

Thus, it does appear that when the apology contains clear statements attesting to controllability and exclusivity, the public is more likely to believe the company’s account and ability to prevent crisis reoccurrence. And thus perhaps counterintuitively, when the organization implicates itself as the source of the wrongdoing, the apology may be better received. This suggests that the public is especially concerned about future events, with control believability implying overall competence and the expectation that it will lead to fewer future problems. Moreover, given that the causal information was communicated by the organization itself, the identification of an internal source may help promote account believability and organizational integrity by demonstrating a willingness of the organization to implicate itself (Blatz et al., 2009; Schumann, 2014; Lewicki et al., 2016). Indeed, given that the identification of an internal and controllable source may also suggest the acceptance of at least some responsibility (even if it was not clearly stated in the scenarios), future research will be needed to clarify the relative influence of causal attributions, especially internal and controllable ones, and responsibility acceptance on apology effectiveness.

Taken together, our results point to the effectiveness of more transparent public statements, and in particular, those that delineate the causal factors and the organization’s ability to control them in the future (Lee et al., 2004). The finding supports the claim that people are generally suspicious of apologies unless they include convincing evidence that the wrongdoer has taken effective actions to rectify matters and prevent future occurrences (Farrell and Rabin, 1996; Coombs and Holladay, 2008; De Cremer, 2010). The results also suggest that causal attributions play a critical role in people’s assessment of the organization involved in the crisis event (Aronson et al., 2010).

### Imaging Findings

To evaluate the public apology, and especially the threat of future reoccurrence, the behavioral evidence suggests that people assess the likelihood that the apologizer will actually control the source of the crisis. To make this control evaluation in response to corporate crises, people must combine two sources of information to obtain a final *control assessment*: actual organizational *controllability* over the crisis source (i.e., whether there is a causal link between them), and the degree of *exclusivity* of control (i.e., whether the organization has relatively exclusive control or whether other external factors could also influence the crisis source). An overall control assessment, in general, appears to be mediated by frontal and ventral prefrontal cortex, with frontal polar cortex activating more for organizational control, and OFC activity higher for external control.

For frontal pole, higher activity in the IC vs. the EU condition appears to reflect processing about the organization, and in particular, the crisis source, the causal link between the organization and the crisis source (i.e., controllability of this internal factor that led to the wrongdoing), and the organization’s wherewithal to control the source (i.e., relative exclusivity). This result thus provides evidence for frontal pole involvement in causal processing. More specifically, assessing the causal link between the organization and the ultimate wrongdoing, and with it, intentionality and responsibility, may entail a degree of mentalizing: e.g., imagining people in the organization and the motivations underlying their actions. Indeed, other imaging studies have also found the frontal pole to be a key region involved in mentalizing and empathy (Farrow et al., 2001; Gilbert et al., 2006; Spreng et al., 2009; Fourie et al., 2017). Nonetheless, whether our findings actually reflect some degree of mentalizing and empathy by our subjects, although suggestive, remains uncertain, requiring examination in future research. Regarding exclusivity, prior evidence appears lacking. Our results, nonetheless, suggest a possible relationship of frontal pole activity not only to individual cause-and-effect relationships but potentially to a comparison and assessment among multiple causes. This finding warrants further research for verification and elaboration, especially as it may relate to other more sophisticated types of relational processing (Bunge et al., 2009).

The OFC finding of higher activity for the EU vs. the IC condition is less clear. It is possible that the activity reflects greater uncertainty and a lack of causal clarity, especially with respect to the degree of control by the organization over the external cause (Hsu et al., 2005). Related to this, the activity may also reflect “other” processing, with less details and perhaps depth of processing about the external source compared to the organization in question (Farrow et al., 2001; Kringelbach and Rolls, 2004; Lieberman, 2007; Spreng et al., 2009; Strang et al., 2014). It is also possible that the activity reflects the greater degree of future threat of reoccurrence, given the organization’s potential lack of control over the external source (Bechara et al., 2000; Kringelbach and Rolls, 2004). Future work will be necessary to isolate the specific factors processed by the OFC related to external causal sources. Indeed, Strang et al. (2014) found greater left OFC activity when receiving an interpersonal apology (as opposed to no apology). The extent to which their results reflect a causality assessment of why the apologizer gave an incorrect answer, or the extent to which ours reflects some degree of believability assessment also require further examination.

For the internal-controllable apology to be effective, it needs to convince the target audience that the apology itself is believable and crisis reoccurrence unlikely. Our results provide evidence that this assessment by the public is mediated by the right angular gyrus. Ohtsubo et al. (2018) also found angular gyrus activation (in particular, the temporal-parietal junction, TPJ, bilaterally) during the assessment of costly apologies (e.g., treating someone to lunch after missing previous appointment as opposed to simply saying sorry), although exactly what was being processed to assess the costly apology remains unclear. Moreover, Strang et al. (2014) found left angular gyrus activation when receiving an apology, and right angular gyrus activation in cases of forgiveness, with forgiveness more likely after a personal apology. Taken together, the results suggest that the angular gyrus underlies both interpersonal and public apology assessment and acceptance.

In addition, our results suggest that the angular gyrus uses causal information to evaluate the apology and potential future threats. Other imaging studies have also found evidence for angular gyrus involvement in both causality (den Ouden et al., 2005; Fugelsang et al., 2005; Seghier, 2013) and threat assessment (Parkinson et al., 2011). Moreover, to evaluate whether the organization can and will intervene and prevent reoccurrence, *interventional* and *counterfactual* causal reasoning is necessary. Thus, our results potentially extend prior findings by suggesting that the angular gyrus also mediates counterfactual causal reasoning.

Others have found evidence for angular gyrus (especially TPJ) involvement in mentalizing and empathy (Gallagher and Frith, 2003; den Ouden et al., 2005; Cavanna and Trimble, 2006; Seghier, 2013; Wende et al., 2013; Jenkins et al., 2014; Strang et al., 2014; Fourie et al., 2017; Jahng et al., 2017). In fact, Ohtsubo et al. (2018) also found TPJ activity for costly interpersonal apologies, suggesting to them that the costly apologies evoked mentalizing and a stronger sense of sincere regret, resulting in greater forgiveness. However, whether the use of causal information to assess possible crisis reoccurrence in our study involved taking the point-of-view of the organization, although possible, is unknown. Future studies are therefore needed to focus on the potential role of mentalizing and related processes such as empathy in causality and threat assessment in the angular gyrus (and frontal pole) as it relates to larger and more complex social groups, such as corporations and the general public, especially given evidence that people may use the same brain regions for mental state attributions of groups (e.g., community groups, corporations) as for individuals (Jenkins et al., 2014).

Although the evidence for frontal pole and angular gyrus involvement found here matches findings in other studies, there were also differences with respect to the entire set of brain regions implicated in our study and others. Differences could potentially result from multiple factors that differ across the studies, especially in methods details (e.g., multiple choice test vs. more realistic or consequential crisis scenarios) and the specific factors tested (e.g., interpersonal vs. public apologies). Further studies are therefore needed to explain these differences. Nonetheless, in sum, our findings provide evidence for frontal pole involvement in causal processing, as well as support other studies that have also found angular gyrus involvement in causal processing, threat, and apology assessment. Our findings may also support those with evidence for mentalizing and empathy by these brain regions. Our study has also extended the previous findings by providing evidence for causal analysis as determined *via* a causal control assessment regarding both controllability and exclusivity in VPFC in general, and for organizational causal control in the frontal pole in particular. We further found evidence for the use of this information to evaluate threats and apologies in the angular gyrus under realistic conditions (news stories of specific organizational crises) and in the context of organizational misdeeds and public apologies.

A complete understanding of the neural mechanisms of social processing requires an examination of progressively more complex social interactions, such as that between organizations and the general public. To our knowledge, our study is only the third to examine the brain regions involved in crisis resolution *via* apologizing (Strang et al., 2014; Ohtsubo et al., 2018) and the first to examine public apologies and the role of causality in intergroup crisis assessment. Future investigations of the neural mechanisms can examine whether such causal determinations (and thus activity in the frontal pole and angular gyrus) underlie other crisis event factors and apology assessments. Future studies can also examine the extent to which actual mentalizing and perspective-taking—i.e., placing oneself in the organization’s position to assess causal control (and other factors)—may underlie public apology assessments (Jenkins et al., 2014). Finally, neuroimaging studies of causal attributions can also look to examine the neural mechanisms of the two key components of causal attribution likelihood: controllability and exclusivity.

A specification of the cognitive and neural mechanisms underlying progressively more complex and abstract social interactions, such as between organizations and the general public during corporate crises, should help to uncover the higher cognitive representations and processing capabilities that evolution, learning and modern society have enabled us to achieve. Our study found that the public appears to assess corporate apology statements based on the details of the crisis to determine how likely the company can correct matters and prevent crisis reoccurrence based on the actual causal factors and the ability to control them. We also have shown how this organizational control assessment is processed in the brain. Indeed, this direction of inquiry—toward greater specificity of the factors and their relationships underlying apology content and its assessment, and in social communication more generally—is necessary to isolate and characterize the fundamental components. Here, we have found that causality appears to be particularly important in people’s assessments and potential forgiveness of wrongdoing. For brain mechanisms, this approach can also be extended not only to teasing apart various factors such as mentalizing and causality assessments but the representations vs. processes involved in each general process as well. We hope our study offers insights for such future investigations.

## Ethics Statement

The Catholic University School of Medicine Review Board and the Korea Advanced Institute of Science and Technology (KAIST) Review Board approved the study.

## Author Contributions

HK and JJ conceived the experimental design. HK, KY, and YC conducted the experiments. HK and JK wrote the main manuscript text. HK, KY and JK prepared the tables and figures. All authors reviewed the manuscript.

## Conflict of Interest Statement

The authors declare that the research was conducted in the absence of any commercial or financial relationships that could be construed as a potential conflict of interest.
