# Supplementary material for: Neural Correlates of Public Apology Effectiveness
Source: Front Hum Neurosci. 2019 Jul 25;13:229. doi: 10.3389/fnhum.2019.00229 (PMC6669883; doi:10.3389/fnhum.2019.00229)
Supplement: Supplementary file 1 [file Table_1.DOCX]

Supporting material

Peaks are reported for all voxel clusters ≥ 10 at p < 0.005. Activations considered statistically significant were those that survived false discovery rate (FDR) correction at p < 0.05 (indicated with *).

Supplementary Table 1. Significant activations in the internal-controllable (IC) condition as contrasted with the external-uncontrollable condition (EU).

| Region | Coordinate | z-score |
| --- | --- | --- |
| Left Frontal Pole (BA 10) | -26, 50, -6 | 3.33* |

Supplementary Table 2. Significant activations in the external-uncontrollable condition (EU) as contrasted with the internal-controllable (IC) condition.

| Region | Coordinate | z-score |
| --- | --- | --- |
| Left Orbitofrontal Cortex (BA 11) | -8, 44, -18 | 3.64* |
| Left Primary Sensory | -33, -33, 57 | 2.2 |
| Right Cerebellum | 21, -51, -24 | 2.11 |

Supplementary Table 3. Significant correlations between regional activity and account acceptance ratings in the internal-controllable condition.

| Region | Coordinate | z-score |
| --- | --- | --- |
| Right Angular Gyrus (BA 39) | 54, -60, 38 | 3.31* |
